# Supplementary material for: Origin of a folded repeat protein from an intrinsically disordered ancestor
Source: eLife. 2016 Sep 13;5:e16761. doi: 10.7554/eLife.16761 (PMC5074805; doi:10.7554/eLife.16761)
Supplement: Supplementary file 1 [file elife-16761-supp1.odt]

Origin of a folded repeat protein from an intrinsically disordered ancestor

Supplementary file 1 

Hongbo Zhu□, Edgardo Sepulveda, Marcus D. Hartmann, Manjunatha Kogenaru†, 
Astrid Ursinus, Eva Sulz, Reinhard Albrecht, Murray Coles, Jörg Martin, Andrei N. Lupas§

Department of Protein Evolution,
Max Planck Institute for Developmental Biology,
Spemannstr. 35, D-72076 Tübingen, Germany

□ hongbo.zhu@tuebingen.mpg.de

† present address: department of life sciences, imperial college london, london sw7 2az, united kingdom. E-mail: m.kogenaru@imperial.ac.uk

 § andrei.lupas@tuebingen.mpg.de; corresponding author

Section A

Table S1. Amino acid variation in RPS20-hh at positions 6, 7, 9 and 23 (TPR repeat unit numbering). The statistics is based on the representative proteomes rp75 sequences (972 sequences) from Pfam family Ribosomal_S20p. If an amino acid is not observed in the position, it is marked NA. Pfam 27.0 was used. 

| Pos\AA | - | A | C | D | E | F | G | H | I | K | L | M | N | P | Q | R | S | T | V | W | Y |
| --- | --- | --- | --- | --- | --- | --- | --- | --- | --- | --- | --- | --- | --- | --- | --- | --- | --- | --- | --- | --- | --- |
| 6 | 4 | 1 | NA | 1 | NA | NA | NA | NA | NA | 686 | NA | NA | 2 | NA | NA | 272 | 4 | 2 | NA | NA | NA |
| 7 | 2 | 114 | NA | 10 | 7 | 1 | 14 | 8 | 5 | 533 | 3 | 1 | 65 | NA | 31 | 118 | 30 | 20 | 10 | NA | NA |
| 9 | 2 | 8 | NA | 31 | 236 | 23 | NA | 15 | 57 | 61 | 99 | 6 | 32 | NA | 22 | 263 | 4 | 3 | 55 | NA | 55 |
| 23 | 5 | 471 | 2 | 6 | 19 | 18 | 6 | 2 | 19 | 25 | 224 | 10 | 13 | 1 | 31 | 18 | 26 | 22 | 42 | 2 | 10 |

Section B

Table S2. Most commonly observed residues at the 34 columns of RPS20 that correspond to the TPR repeat unit. The statistics is based on the representative proteomes rp75 sequences (972 sequences) from Pfam 27.0 family Ribosomal_S20p.

Position	1st		2nd		3rd			KR  		CDEHKNQRSTWY

1		L (300, 30.9%)	M (225, 23.1%)	V (202, 20.8%)		 0.0%		 0.0%

2		R (640, 65.8%)	K (303, 31.2%)	H ( 10,  1.0%)		97.0%		98.0%	

3		T (790, 81.3%)	N ( 95,  9.8%)	S ( 32,  3.3%)		 0.0%		94.3%	

4		A (389, 40.0%)	F (166, 17.1%)	Y (124, 12.8%)		 0.0%		12.8%

5		I (449, 46.2%)	V (293, 30.1%)	L ( 70,  7.2%)		 0.0%		 0.0%

6		K (686, 70.6%)	R (272, 28.0%)	S (  4,  0.4%)		98.6%		99.0%	

7		K (533, 54.8%)	R (118, 12.1%)	A (114, 11.7%)		67.0%		67.0%	

8		V (465, 47.8%)	F (230, 23.7%)	L ( 78,  8.0%)		 0.0%		 0.0%

9		R (263, 27.1%)	E (236, 24.3%)	L ( 99, 10.2%)		27.1%		51.3%	

10		E (239, 24.6%)	A (239, 24.6%)	K (179, 18.4%)		18.4%		43.0%

11		A (748, 77.0%)	L ( 50,  5.1%)	S ( 29,  3.0%)		 0.0%		 3.0%

12		I (362, 37.2%)	V (276, 28.4%)	L (127, 13.1%)		 0.0%		 0.0%

13		A (299, 30.8%)	E (241, 24.8%)	D ( 85,  8.7%)		 0.0%		33.5%

14		A (396, 40.7%)	S (155, 15.9%)	T ( 84,  8.6%)		 0.0%		24.6%

15		G (569, 58.5%)	K (103, 10.6%)	N ( 86,  8.8%)		10.6%		19.4%

16		D (606, 62.3%)	N (111, 11.4%)	A ( 58,  6.0%)		 0.0%		73.8%	

17		K (425, 43.7%)	A (116, 11.9%)	V ( 76,  7.8%)		43.7%		43.7%

18		E (321, 33.0%)	A (234, 24.1%)	D (119, 12.2%)		 0.0%		45.3%

19		A (293, 30.1%)	K (164, 16.9%)	E (124, 12.8%)		16.9%		29.6%

20		A (768, 79.0%)	V ( 62,  6.4%)	S ( 33,  3.4%)		 0.0%		 3.4%

21		Q (178, 18.3%)	E (168, 17.3%)	T (117, 12.0%)		 0.0%		47.6%

22		E (281, 28.9%)	A (260, 26.7%)	K (110, 11.3%)		11.3%		40.2%

23		A (471, 48.5%)	L (224, 23.0%)	V ( 42,  4.3%)		 0.0%		 0.0%

24		L (368, 37.9%)	F (309, 31.8%)	Y (131, 13.5%)		 0.0%		13.5%

25		K (199, 20.5%)	R (189, 19.4%)	A (106, 10.9%)		39.9%		39.9%

26		A (223, 22.9%)	E (144, 14.8%)	K (116, 11.9%)		11.9%		26.7%

27		A (666, 68.5%)	V (106, 10.9%)	M ( 68,  7.0%)		 0.0%		 0.0%

28		Q (223, 22.9%)	S (147, 15.1%)	V (137, 14.1%)		 0.0%		38.1%

29		S (309, 31.8%)	P (236, 24.3%)	K (217, 22.3%)		22.3%		54.1%	

30		K (180, 18.5%)	V (144, 14.8%)	E (120, 12.3%)		18.5%		30.9%

31		L (433, 44.5%)	I (382, 39.3%)	V (111, 11.4%)		 0.0%		 0.0%

32		D (781, 80.3%)	M ( 72,  7.4%)	H ( 35,  3.6%)		 0.0%		84.0%	

33		K (482, 49.6%)	R (318, 32.7%)	S ( 49,  5.0%)		82.3%		87.3%	

34		A (525, 54.0%)	M ( 80,  8.2%)	L ( 78,  8.0%)		 0.0%		 0.0%

Section C 

Table S3. List of putative TPR homologs identified by sequence and structure analysis. See Method section in the article for details.  

Section D

Table S4. TPRpred hit list by scanning a merged sequence dataset of ncbi and full from Pfam family Ribosomal_S20p (PF01649). A per-repeat P-value cutoff of 1e-4 is used. 

>218294917/8-90(26-59)         Len=83  E-prot=2.01e-03 P-rep=5.4e-07

IKTLSKKAVLLAQEGKAEEAIKIMRKAVSLIDKA

>53803711/2-84(27-60)         Len=83  E-prot=1.72e-02 P-rep=4.6e-06

LRTYIKKVILAVDSGDLTKAQEAFRQAVPIIDSS

>315121827/2-84(27-60)         Len=83  E-prot=2.18e-02 P-rep=5.8e-06

VRTFIRRANEAIAVGKVEEATEACKKAESLVQKA

>358448474/2-84(27-60)         Len=83  E-prot=2.39e-02 P-rep=6.4e-06

ARTYVKKIQSKIEAGNYEEAQAAFQQAQPILDSM

>384439015/9-90(25-58)         Len=82  E-prot=3.21e-02 P-rep=8.6e-06

IKTLSKKAVQLAQEGQAEEAIKILRKAESLIDKA

>149377785/2-84(27-60)         Len=83  E-prot=8.55e-02 P-rep=2.3e-05

ARTYIKKVQANIEAGKPEEAQAALQKAQPIMDSM

>120553791/2-84(27-60)         Len=83  E-prot=1.07e-01 P-rep=2.9e-05

ARTYMKKVDAKIKAGNHDEAQAALKEAQPIMDSM

>358011912/2-84(27-60)         Len=83  E-prot=1.12e-01 P-rep=3.0e-05

VRTYIKRTVAAIAAGDYTVATDAYKKAVPVIDRM

>389711173/2-84(27-60)         Len=83  E-prot=1.19e-01 P-rep=3.2e-05

VRTYLKRTVAAIAAGDYAVATEAYKKAVPVIDRM

>71066042/2-84(27-60)         Len=83  E-prot=1.20e-01 P-rep=3.2e-05

VRTYLKRVDAAIAAKDYDAATEAYKKAVPVLDRM

>320451144/9-90(25-58)         Len=82  E-prot=1.44e-01 P-rep=3.9e-05

IKTLSKKAVLLAQEGKAEEALKIMRMAQSLIDKA

>34811548/2-82(25-58)         Len=81  E-prot=1.51e-01 P-rep=4.0e-05

IKTLSKKAVQLAQEGKAEEALKIMRKAESLIDKA

>126666692/2-84(27-60)         Len=83  E-prot=1.70e-01 P-rep=4.5e-05

ARTYMKKIHVQIAEGNYEGAKAAFLQAQPILDSM

>297565393/9-90(25-58)         Len=82  E-prot=1.81e-01 P-rep=4.8e-05

IKTISKKAVALAQEGNTEEATKFLRLAESLIDKA

>295698740/2-81(24-57)         Len=80  E-prot=1.86e-01 P-rep=5.0e-05

IKTFTKRVLSFLSNKDIEQAKYYFNRVQSILDRF

>224510696/2-82(25-58)         Len=81  E-prot=1.97e-01 P-rep=5.3e-05

IKTLSKKAIQLAQEGKAEEALKIMRKAESLIDKA

>254429490/2-61(4-37)         Len=60  E-prot=2.14e-01 P-rep=5.7e-05

VRTYLKKVNAAIASGDQGSAQEAYNQAVSVLDKA

>255318942/2-84(27-60)         Len=83  E-prot=2.27e-01 P-rep=6.1e-05

VRTYIKRTLSAIAAGDYAVATEAYKKAVPVIDRM

>83648579/2-84(27-60)         Len=83  E-prot=2.56e-01 P-rep=6.8e-05

VRTYIKKVSAQIEQGSYDGATQALTTAAPIIDSM

>148652754/2-84(27-60)         Len=83  E-prot=2.58e-01 P-rep=6.9e-05

IRTYIKKVDAAILAGDYDAATAAYNKAVPVIDRM

>160872516/4-85(27-60)         Len=82  E-prot=2.60e-01 P-rep=7.0e-05

MRTYIKRVVVAIEAGEATKAKDAYQTALPIIDRM

>50084567/2-84(27-60)         Len=83  E-prot=2.93e-01 P-rep=7.8e-05

VRTYIKRTVNAIAAGDYTVATEAYKKAVPVIDRM

>333367735/2-84(27-60)         Len=83  E-prot=2.95e-01 P-rep=7.9e-05

IRTYIKKVDAAILAGDYEAATAAYNKAVPVIDRM

>126641662/2-84(27-60)         Len=83  E-prot=3.56e-01 P-rep=9.5e-05

VRTYIKRTLSAIAGGDYAVATEAYKKAVPVIDRM

Section E

Table S5. The mutation combinations tested in silico. 

K7 was found to co-evolve with I23 (see article). Therefore, they were considered to be a single mutation and was mutated together. I23 was also mutated independently because K7_I23D is physiochemically similar to K7R_I23D.

1) Primary mutations are defined to be mutations introduced following the principle of census design. 
2) Secondary mutations are defined to be mutations introduced to lower protein pI values.

| Primary mutations 1) | L4W, K7L_I23Y, K7R_I23D, V9N, I23D |
| --- | --- |
| Secondary mutations 2) | K2E, K6N, K22E, R25E, R25Q |
| All combinations of primary mutations (15) | Single (5): Double (7): Triple (3): | L4W, K7L_I23Y, K7R_I23D, V9N, I23D L4W_K7L_I23Y, L4W_V9N, l4W_I23D, L4W_K7R_I23D, K7L_V9N_I23Y, V9N_I23D, K7R_V9N_I23D L4W_K7L_V9N_I23Y, L4W_V9N_I23D, L4W_K7R_V9N_I23D |
| Tested combinations involving both primary and secondary mutations (25) | K2E_K7L_I23Y, K6N_K7L_I23Y, K7L_K22E_I23Y, K7L_I23Y_R25Q, K7L_I23Y_R25E, K2E_K7L_V9N_I23Y, K6N_K7L_V9N_I23Y, K7L_V9N_K22E_I23Y, K7L_V9N_I23Y_R25Q, K7L_V9N_I23Y_R25E, K2E_L4W_K7L_V9N_I23Y, L4W_K6N_K7L_V9N_I23Y, L4W_K7L_V9N_K22E_I23Y, L4W_K7L_V9N_I23Y_R25Q, L4W_K7L_V9N_I23Y_R25E, K2E_K7R_V9N_I23D, K6N_K7R_V9N_I23D, K7R_V9N_K22E_I23D, K7R_V9N_I23D_R25Q, K7R_V9N_I23D_R25E, K2E_L4W_K7R_V9N_I23D, L4W_K6N_K7R_V9N_I23D, L4W_K7R_V9N_K22E_I23D, L4W_K7R_V9N_I23D_R25Q, L4W_K7R_V9N_I23D_R25E |

The fixbb command line:

fixbb.linuxgccrelease -s input_pdb -use_input_sc -resfile resfile_mut -ex1 -ex2 -minimize_sidechains -ndruns 10 -nstruct 1000

The relax command line:

relax.linuxgccrelease -relax:fast -ex1 -ex2 -ndruns 10 -nstruct 10000

Section F

Table S6. Biophysical parameters of designed TPR repeats. Folded proteins yielding a non-cooperative thermal melting curve are marked by an asterisk (*). Urea denaturation parameters are ∆GU-FH2O, the free energy change in the absence of denaturant; Cm, the urea concentration at the curve midpoint; and mU-F, the sensitivity of the transition to denaturant.

|  | Circular dichroism | Urea denaturation | Static light scattering |  |
| --- | --- | --- | --- | --- |
|  | a-helical lmin 222 nm | Tm °C | ∆GU-F H2O  kcal·mol-1 | mU-F kcal·mol-1 M-1 | Cm M | Calculated MW kDa | Monomer MW kDa | Folded  protein |
| M0 | - | - | - | - | - | 16.4 | 14.4 | - |
| M2 | + | -* | 4.38 ± 0.19 | 1.25 ± 0.05 | 3.50 | 27.4 | 14.5 | + |
| M4E | + | -* | 7.87 ± 0.29 | 1.38 ± 0.05 | 5.67 | 31.0 | 14.6 | + |
| M4N | + | 77 | 5.78 ± 0.13 | 1.36 ± 0.03 | 4.27 | 20.9 | 14.5 | + |
| M4N∆C | + | 78 | 5.43 ± 0.14 | 1.22 ± 0.03 | 4.46 | 20.1 | 11.9 | + |
| M4N-His | + | 75 | 4.80 ± 0.21 | 1.15 ± 0.05 | 4.17 | 28.7 | 15.4 | + |
| M4RD | - | - | - | - | - | 15.3 | 14.4 | - |
| M5 | + | -* | 2.81 ± 0.07 | 0.81 ± 0.06 | 3.49 | 27.8 | 14.5 | + |
| Rps20 T.a. | - | - | - | - | - | 9.8 | 10.8 | ± |
| Rps20 T.t. | - | - | - | - | - | 9.9 | 11.7 | ± |

Section G

Table S7. The primary structures of polypeptide chains in the ASUs of M4N (three chains) and the two crystal forms of M4N∆C: M4N∆C CF I (four chains) and M4N∆C CF II (one chain).

>M4N chain A
NS
IKTLSNLANLLAQEGKAEEAIKYMRKAVSLDPNN
IKTLSNLANLLAQEGKAEEAIKYMRKAVSLDPNN
IKTLSNLAVLLAQEGKAEEAIKYMRKAVSLIDKA
AK

>M4N chain B
NS
IKTLSNLANLLAQEGKAEEAIKYMRKAVSLDPNN
IKTLSNLANLLAQEGKAEEAIKYMRKAVSLDPNN
IKTLSNLAVLLAQEGKAEEAIKYMRKAVSLIDKA
A

>M4N chain C
GNS
IKTLSNLANLLAQEGKAEEAIKYMRKAVSLDPNN
IKTLSNLANLLAQEGKAEEAIKYMRKAVSLDPNN
IKTLSNLAVLLAQEGKAEEAIKYMRKAVSLIDKA
AK

>M4NΔC CF I chain A 
NS
IKTLSNLANLLAQEGKAEEAIKYMRKAVSLDPNN
IKTLSNLANLLAQEGKAEEAIKYMRKAVSLDPNN
IKTLSNLAVLLAQEGKAEEAIKYMRKAVSLIDKA
AKG

>M4NΔC CF I chain B
NS
IKTLSNLANLLAQEGKAEEAIKYMRKAVSLDPNN
IKTLSNLANLLAQEGKAEEAIKYMRKAVSLDPNN
IKTLSNLAVLLAQEGKAEEAIKYMRKAVSLIDKA
A

>M4NΔC CF I chain C
NS
IKTLSNLANLLAQEGKAEEAIKYMRKAVSLDPNN
IKTLSNLANLLAQEGKAEEAIKYMRKAVSLDPNN
IKTLSNLAVLLAQEGKAEEAIKYMRKAVSLIDKA
AKG

>M4NΔC CF I chain D
NS
IKTLSNLANLLAQEGKAEEAIKYMRKAVSLDPNN
IKTLSNLANLLAQEGKAEEAIKYMRKAVSLDPNN
IKTLSNLAVLLAQEGKAEEAIKYMRKAVSLIDKA
A

>M4NΔC CF II chain A
NS
IKTLSNLANLLAQQGKAEEAIKYMRKAVSLDPNN
IKTLSNLANLLAQEGKAEEAIKYMRKAVSLDPNN
IKTLSNLAVLLAQEGKAEEAIKYMRKAVSLIDKA
AKG

Section H

Table S8. Crystallization and cryo conditions.

| Structure | Reservoir solution (RS) | Cryo solution |
| --- | --- | --- |
| M4N | 200 mM lithium sulfate; 20% PEG 3350 | RS + 20% PEG 400 |
| M4NΔC CF I | 100 mM Tris HCl pH 8.5; 30% PEG 400 | - |
| M4NΔC CF II | 100 mM SPG(*) pH 8; 25% PEG 1500 | RS + 15% glycerol |

(*) SPG Buffer as formulated in the QUIAGEN PACT screen. 

Table S9. Data collection and refinement statistics

|  | M4N | M4NΔC |
| --- | --- | --- |
| Native | K2PtCl4 derivative | Native, CF I | Native, CF II |
| Wavelength (Å) | 1.0 | 1.071 | 1.071 | 1.0 |
| Space group | P3121 | P3121 | P21 | P622 |
| Cell dimensions | a=b=92.23 Å, c=76.41 Å | a=b=94.39 Å, c=76.51 Å | a=49.08 Å, b=50.31 Å, c=83.10 Å, β=102.4° | a=b=64.75 Å, c=86.83 Å |
| Monomers / ASU | 3 | 3 | 4 | 1 |
| Resolution (Å) | 35.4 - 2.15 (2.28 – 2.15) | 36.1 - 2.50 (2.66 – 2.50) | 38.3 - 2.05 (2.17 - 2.05) | 34.3 - 1.65 (1.75 - 1.65) |
| Completeness (%) | 98.6 (97.4) | 98.6 (95.0) | 98.4 (94.1) | 99.6 (99.0) |
| Redundancy | 4.38 (4.13) | 13.9 (13.9) | 5.58 (5.69) | 9.21 (9.44) |
| I/σ(I) | 15.51 (2.54) | 19.32 (3.40) | 18.4 (2.10) | 32.6 (3.28) |
| Rmerge (%) | 6.5 (69.7) | 10.6 (94.1) | 5.5 (70.8) | 3.9 (71.3) |
| Rcryst / Rfree (%) | 22.7 / 26.4 |  | 21.8 / 26.5 | 19.8 / 21.9 |
| RMSD Bond angles/lengths | 0.797/0.005 |  | 0.811/0.005 | 0.967/0.007 |
| Ramachandran plot statistics (%) | 94.3 / 5.7 / 0 |  | 94.4 / 5.6 / 0 | 95 / 5 / 0 |
| PDB code | 5FZQ |  | 5FZR | 5FZS |

Values in parentheses refer to the highest resolution shell. The Ramachandran plot statistics show the percentage of residues in the most favored / additionally allowed / other regions, respectively, as defined and determined using the program PROCHECK.

Section I

Table S10. RMSD between the three polypeptide chains in the ASU of M4N (PDB id: 5fzq) after superposition. Numbers of atoms considered for the superposition and calculation of RMSD are given in parentheses. There are 105 residues in common among the three chains (residues 1-105). Three RMSD values are calculated for each pair of chains: all atoms (variable numbers of atoms) / Cα of all common residues (105 atoms) / Cα of the three TPR units (102 atoms).

|  | Chain A | Chain B |
| --- | --- | --- |
| Chain B | 1.64 (771) / 1.29 (105) / 1.30 (102) | - |
| Chain C | 1.28 (738)  / 0.93 (105) / 0.88 (102) | 1.61 (729) / 1.35 (105) / 1.32 (102) |

Table S11. Cα RMSD between M4N (PDB id: 5fzq) and CTPR3 (PDB: 1na0, chain A) after superposition. Numbers of atoms considered for the superposition and calculation of RMSD are given in parentheses. Only the first two TPR units and the first 30 residues of the third TPR units are considered ( 34 + 34 + 30 = 98 residues ).

|  | M4N_A | M4N_B | M4N_C |
| --- | --- | --- | --- |
| CTPR3 (1na0_A) | 2.57 (98) | 1.93 (98) | 2.33 (98) |

Table S12. Cα RMSD between  M4NΔC CF I (crystal form with four chains in the ASU, PDB id: 5fzr) and CTPR3 after superposition. Numbers of atoms considered for the superposition and calculation of RMSD are given in parentheses. 

|  | M4NΔC CF I_A | M4NΔC CF I_B | M4NΔC CF I_C | M4NΔC CF I_D |
| --- | --- | --- | --- | --- |
| CTPR3 (1na0_A) | 2.72 (98) | 1.95 (98) | 2.69 (98) | 2.11 (98) |
| M4NΔC CF I_B | 1.21 (102) | - | - | - |
| M4NΔC CF I_C | 0.44 (102) | 1.10 (102) | - | - |
| M4NΔC CF I_D | 1.13 (102) | 0.41 (102) | 1.00 (102) | - |

Table S13. Cα RMSD between M4NΔC CF II (crystal form with only one chain in the ASU, PDB id: 5fzs) and CTPR3 after superposition. Numbers of atoms considered for the superposition and calculation of RMSD are given in parentheses. 

|  | M4NΔC CF II_A |
| --- | --- |
| CTPR3 (1na0_A) | 2.72 (98) |

Table S14. Cα RMSD between dimeric complexes in M4N,  M4NΔC CF I and  M4NΔC CF II. Numbers of atoms considered for the superposition and calculation of RMSD are given in parentheses. 

|  | M4NΔC CF I_AB | M4NΔC CF I_CD | M4NΔC CF II_AB |
| --- | --- | --- | --- |
| M4N | 0.59 (204) | 0.78 (204) | 1.82 (204) |
| M4NΔC CF I_AB | - | 0.55 (204) | 1.81 (204) |
| M4NΔC CF I_CD | - | - | 1.63 (204) |

Table S15. RMSD between the three TPR units in the three chains of M4N (PDB: 5fzq). Numbers of atoms considered for the superposition and calculation of RMSD are given in parentheses. Only the first 30 residues in the third TPR unit are considered.

| M4N_A | TPR unit 1 | TPR unit 2 |
| --- | --- | --- |
| TPR unit 2 | 1.15 (257) / 0.42 (34) | - |
| TPR unit 3 | 1.00 (218) / 0.40 (30) | 0.87 (214) / 0.31 (30) |

| M4N_B | TPR unit 1 | TPR unit 2 |
| --- | --- | --- |
| TPR unit 2 | 1.27 (250) / 0.59 (34) | - |
| TPR unit 3 | 1.18 (210) / 0.43 (30) | 0.90 (221) / 0.35 (30) |

| M4N_C | TPR unit 1 | TPR unit 2 |
| --- | --- | --- |
| TPR unit 2 | 0.97 (231) / 0.65 (34) | - |
| TPR unit 3 | 0.99 (206) / 0.66 (30) | 0.82 (195) / 0.31 (30) |

Section J

Table S16. SEG prediction results of low-complexity regions in the ribosomal protein S20 of Thermus aquaticus (NCBI ACCESSION: WP_003044315.1;  GI: 489134531). The helical hairpin RPS20-hhta is underlined (residue 33-66).

| SEG parameters | SEG output |
| --- | --- |
| Low-complexity segments                                  High-complexity segments |
| -window 12  -locut 2.2  -hicut 2.5 | 1-97  MATKKPKKNLSALKRHRQSLKRRLRNKAKKSAIKT                                             LSKKAVLLAQEGKAEEAIKIMRKAVSLIDKAAKGS                                             TLHKNAAARRKSRLMRKVQKLLSAVSA |
| -window 25  -locut 3.0  -hicut 3.3 | 1-3   MAT  kkpkknlsalkrhrqslkrrlrnkakksaiktlsk  4-62               kavllaqegkaeeaikimrkavsl                                      63-97  IDKAAKGSTLHKNAAARRKSRLMRKVQKLLSAVSA |
| -window 45  -locut 3.4  -hicut 3.75 | matkkpkknlsalkrhrqslkrrlrnkakksaikt  1-97    lskkavllaqegkaeeaikimrkavslidkaakgs          tlhknaaarrksrlmrkvqkllsavsa |
